# Association Between Graves’ Disease and the Risk of Thyroid Carcinoma: The Role of Clinical, Metabolic, Hormonal, Immunological, and Ultrasound Biomarkers

**DOI:** 10.3390/metabo16080580

**Published:** 2026-08-17

**Authors:** Mihaela Andreea Precup, Andrei Korodi, Flaviu Ionut Faur, Paul Pasca, Draga-Maria Mandi, Dan Brebu, Cosmin Burta, Amadeus Dobrescu, Ciprian Duta

**Affiliations:** 1 Doctoral School, Victor Babes University of Medicine and Pharmacy Timisoara, E. Murgu Square, No. 2, 300041 Timisoara, Romania; mihaela.precup@umft.ro (M.A.P.); paul.pasca@umft.ro (P.P.); mihai.burta@umft.ro (C.B.); 2 2nd Surgery Clinic, Emergency Clinical County Hospital of Arad, 2-4 Andreny Karoly Str., 310037 Arad, Romania; 3 Department of Medicine, Faculty of Medicine, “Vasile Goldis” Western University of Arad, L. Rebreanu St. 86, 310048 Arad, Romania; 4 Researching Future Surgery II Research Center, Department X, Discipline of General Surgery II, Faculty of Medicine, Victor Babes University of Medicine and Pharmacy Timisoara, E. Murgu Square, No. 2, 300041 Timisoara, Romania; brebu.dan@umft.ro (D.B.); dobrescu.amadeus@umft.ro (A.D.); duta.ciprian@umft.ro (C.D.); 5 General Surgery Department, Coltea Clinical Hospital, Bulevardul Ion C. Brătianu 1, 030171 București, Romania

**Keywords:** Graves’ disease, thyroid carcinoma, papillary thyroid carcinoma, biomarkers, thyroid ultrasound, metabolic biomarkers, lipid profile, TRAb, systematic review, meta-analysis, PRISMA 2020

## Abstract

Background: The relationship between Graves’ disease (GD) and thyroid carcinoma (TC) remains controversial, particularly regarding the predictive value of metabolic, hormonal, immunological, and ultrasound biomarkers. We performed a systematic review and meta-analysis to evaluate the prevalence of TC in patients with GD and to identify biomarkers associated with an increased risk of malignancy. Methods: A systematic literature search of PubMed/MEDLINE, Scopus, Web of Science, Embase, and the Cochrane Library was conducted in accordance with PRISMA 2020. Primary studies providing direct evidence in patients with Graves’ disease or indirect evidence concerning thyroid carcinoma-associated biomarkers were considered. Owing to substantial differences in study populations, designs, exposures, and outcomes, quantitative pooling was restricted to sufficiently comparable estimates, while the remaining evidence was synthesized descriptively. Results: Six primary studies were included. Two retrospective cohorts directly evaluated thyroid carcinoma in patients with Graves’ disease and included 1051 patients, of whom 317 had thyroid carcinoma. The remaining studies provided indirect evidence from patients with hyperthyroidism, population-based thyroid cancer cohorts, or genetic datasets. Because of substantial clinical and methodological heterogeneity, a single pooled estimate was not considered appropriate. Thyroid nodules and larger nodule size were associated with malignancy in Graves’ disease, while elevated triglycerides, reduced HDL cholesterol, and higher body mass index were associated with thyroid carcinoma in a surgically selected Graves’ disease cohort. Evidence concerning hormonal, immunological, inflammatory, and genetically determined metabolic markers was heterogeneous and largely indirect. Conclusions: Thyroid carcinoma risk in Graves’ disease is heterogeneous and appears to be concentrated in patients presenting with suspicious ultrasound findings and adverse metabolic profiles. Ultrasound biomarkers remain the most robust predictors of malignancy, while metabolic biomarkers represent promising complementary tools for individualized risk stratification. Current evidence does not support the routine use of hormonal or immunological biomarkers as independent predictors of thyroid carcinoma. Large prospective multicenter studies using standardized biomarker assessment are required to validate these findings and optimize personalized surveillance strategies.

## 1. Introduction

Graves’ disease (GD) is the most common cause of autoimmune hyperthyroidism, affecting approximately 1–2% of the general population and predominantly occurring in women [1,2,3]. The disease is characterized by the production of thyroid-stimulating hormone receptor antibodies (TRAbs), which stimulate thyroid hormone synthesis and secretion, leading to diffuse goiter and thyrotoxicosis. Beyond its endocrine manifestations, Graves’ disease has attracted increasing attention because of its potential association with thyroid malignancies, particularly differentiated thyroid carcinoma (DTC), including papillary thyroid carcinoma (PTC), the most common histological subtype of thyroid cancer [4,5,6,7,8].

The relationship between Graves’ disease and thyroid carcinoma remains controversial. Several epidemiological studies have reported a higher prevalence of thyroid nodules and thyroid cancer among patients with Graves’ disease compared with the general population. Proposed mechanisms include chronic autoimmune stimulation, sustained activation of the thyroid-stimulating hormone receptor pathway, increased angiogenesis, oxidative stress, and persistent inflammatory responses within the thyroid microenvironment. Conversely, other studies have failed to demonstrate a significant increase in thyroid cancer risk attributable to Graves’ disease itself, suggesting that the observed association may be mediated by concomitant thyroid nodules or other clinical risk factors [9,10,11].

In recent years, considerable effort has been directed toward identifying biomarkers capable of improving risk stratification in patients with Graves’ disease. Clinical and ultrasound characteristics, such as nodule size, multiplicity, microcalcifications, irregular margins, and hypoechogenicity, have been associated with an increased likelihood of malignancy. Furthermore, metabolic biomarkers, including triglycerides, high-density lipoprotein (HDL) cholesterol levels, and body mass index (BMI), have emerged as potential predictors of thyroid carcinoma development. At the same time, the prognostic value of hormonal and immunological markers, including thyroid-stimulating hormone (TSH), free thyroxine (FT4), free triiodothyronine (FT3), and TRAbs, remains inconsistent across studies [12,13,14,15,16].

The growing interest in inflammation and cancer biology has further highlighted the importance of the immune microenvironment in thyroid tumorigenesis [17,18,19,20]. Autoimmune thyroid disorders may influence carcinogenesis through complex interactions between inflammatory cytokines, immune cell infiltration, and molecular signaling pathways [21,22,23,24,25,26,27]. Consequently, identifying reliable biomarkers capable of distinguishing Graves’ disease patients at higher risk of thyroid carcinoma could facilitate earlier diagnosis, individualized surveillance strategies, and optimized therapeutic decision-making [28,29,30,31,32,33,34,35]. Despite the increasing number of studies investigating this association, the available evidence remains heterogeneous, and no consensus currently exists regarding the most clinically relevant biomarkers for predicting thyroid carcinoma in patients with Graves’ disease. Therefore, a comprehensive synthesis of the current literature is warranted [36,37,38,39,40,41,42,43].

Unlike previous reviews that primarily focused on the prevalence of thyroid carcinoma in Graves’ disease or on isolated clinical risk factors, the present study provides a comprehensive synthesis of multiple biomarker domains, including clinical, metabolic, hormonal, immunological, inflammatory, and ultrasound-derived biomarkers associated with thyroid carcinoma. By integrating evidence across these complementary biomarker categories, this review aims to identify clinically applicable predictors that may improve individualized risk stratification, optimize surveillance strategies, and support earlier detection of thyroid carcinoma in patients with Graves’ disease.

The aim of this systematic review and meta-analysis was to evaluate the association between Graves’ disease and thyroid carcinoma risk and to assess the predictive value of clinical, metabolic, hormonal, immunological, and ultrasound biomarkers for the identification of patients at increased risk of thyroid malignancy.

## 2. Materials and Methods

### 2.1. Study Design and Reporting Guidelines

This study was designed as a systematic review and meta-analysis to evaluate biomarkers associated with thyroid carcinoma in patients with Graves’ disease. The review focused on identifying and critically appraising available evidence regarding clinical, biochemical, immunological, inflammatory, imaging, and molecular predictors that may assist in estimating the risk of thyroid malignancy in this specific patient population. The review methodology was developed in accordance with the Preferred Reporting Items for Systematic Reviews and Meta-Analyses (PRISMA 2020) statement, and all stages of study selection, data extraction, quality assessment, and evidence synthesis were performed using predefined eligibility criteria. Because of the heterogeneity of the available literature, quantitative synthesis was performed only when studies reported sufficiently comparable outcomes and effect estimates; otherwise, findings were synthesized narratively.

### 2.2. Literature Search Strategy

A comprehensive literature search was undertaken to identify studies evaluating biomarkers associated with thyroid carcinoma in patients with Graves’ disease. Searches were conducted in PubMed/MEDLINE, Scopus, Web of Science, Embase, and the Cochrane Library using combinations of controlled vocabulary and free-text keywords related to Graves’ disease, thyroid carcinoma, and biomarkers associated with thyroid carcinoma. The search strategy was developed before study selection and applied consistently across all databases, with database-specific adaptations where required. The final search was completed on 19 March 2026. No language restrictions were applied during the literature search. This systematic review was not prospectively registered in PROSPERO or any other international review registry.

The search strategy combined Medical Subject Headings (MeSH) terms and free-text keywords related to Graves’ disease, thyroid carcinoma, and biomarkers. The following search terms were used in different combinations: (“Graves disease” OR “autoimmune hyperthyroidism”) AND (“thyroid cancer” OR “thyroid carcinoma” OR “papillary thyroid carcinoma” OR “differentiated thyroid carcinoma”) AND (“biomarker” OR “thyroid stimulating hormone receptor antibody” OR “TRAb” OR “TSH” OR “FT4” OR “FT3” OR “thyroid nodule” OR “ultrasound” OR “lipid profile” OR “HDL cholesterol” OR “triglycerides” OR “body mass index”). Additionally, the reference lists of eligible studies and relevant review articles were manually screened to identify potentially relevant publications not retrieved during the electronic search. The complete electronic search strategy used for the PubMed/MEDLINE database is provided in Supplementary Material S1 to facilitate transparency and reproducibility.

### 2.3. Eligibility Criteria

Studies were considered eligible if they evaluated adult patients with Graves’ disease and investigated clinical, metabolic, hormonal, immunological, inflammatory, ultrasound, or molecular biomarkers associated with thyroid carcinoma. Observational studies were also considered eligible, as each study design provided complementary evidence regarding the association between Graves’ disease and thyroid carcinoma. Previously published systematic reviews and meta-analyses were screened to identify additional relevant primary studies and to contextualize the findings but were not considered eligible for evidence synthesis or quantitative analysis.

Studies were excluded if they consisted of case reports, case series, conference abstracts, editorials, narrative reviews, letters to the editor, animal or in vitro studies, or if they did not evaluate biomarkers relevant to thyroid carcinoma in patients with Graves’ disease. Duplicate publications, studies with overlapping populations, and studies lacking sufficient methodological or outcome data were also excluded.

#### 2.3.1. Inclusion Criteria

Studies were considered eligible if they evaluated adult patients with Graves’ disease and investigated clinical, laboratory, imaging, inflammatory, immunological, molecular, or metabolic biomarkers associated with the presence or risk of thyroid carcinoma.

#### 2.3.2. Exclusion Criteria

Conference abstracts, editorials, letters, narrative reviews, case reports, animal studies, and studies lacking sufficient methodological or outcome data were excluded. Publications that did not specifically evaluate biomarkers relevant to thyroid carcinoma in patients with Graves’ disease or that provided duplicate datasets were also excluded.

### 2.4. Study Selection

**Figure 1:**
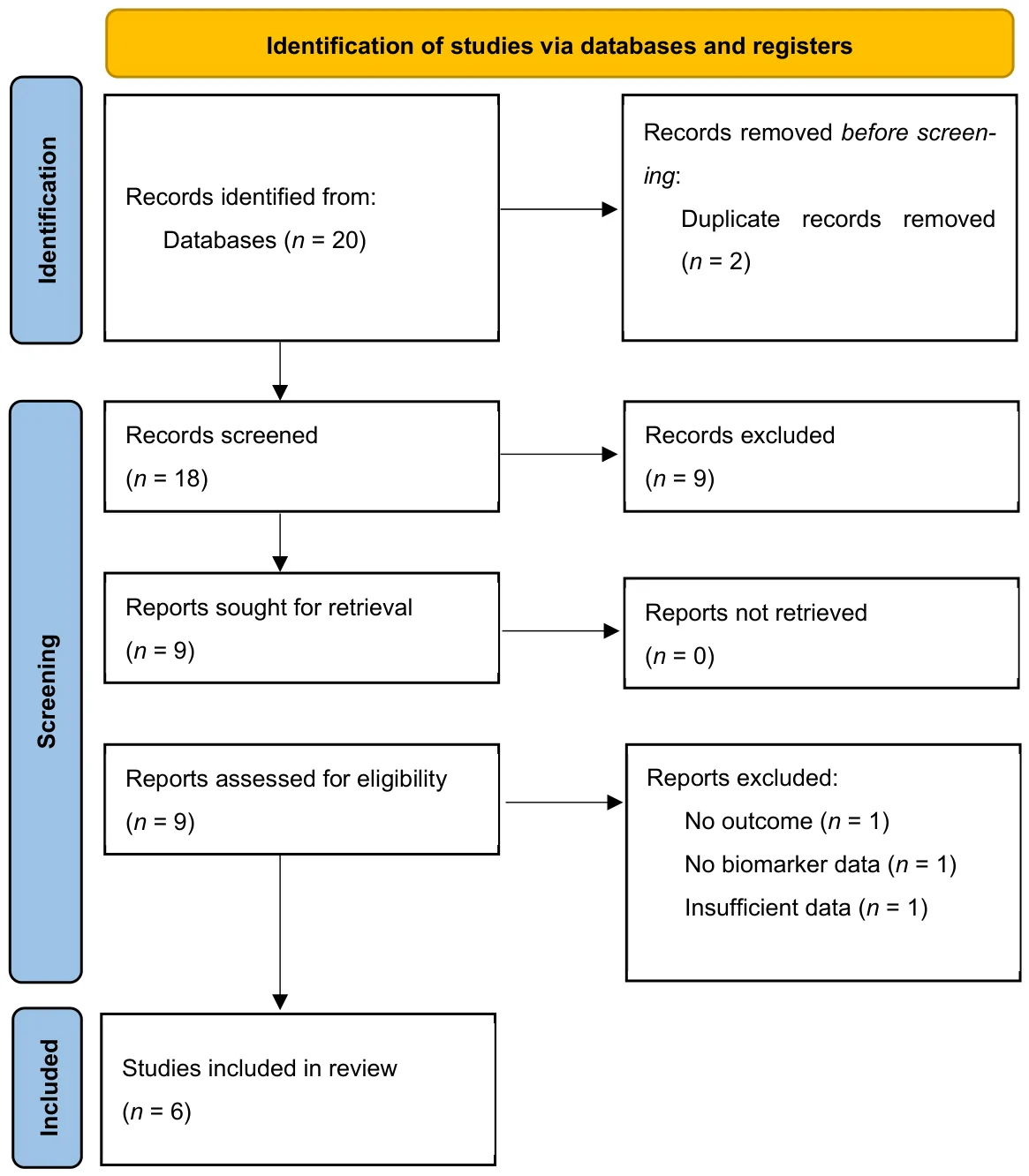
PRISMA 2020 flow diagram of the study selection process. The diagram illustrates the identification, screening, eligibility assessment, and inclusion of studies investigating the association between Graves’ disease and thyroid carcinoma risk based on clinical, biochemical, immunological, and imaging biomarkers.

All records identified through database searching were imported into a reference management software and screened for duplicates. Following database searching, all retrieved records were imported into the reference management software and screened for duplicate entries. Two reviewers independently assessed titles and abstracts according to the predefined eligibility criteria. Potentially relevant articles subsequently underwent full-text evaluation, with any disagreement resolved through discussion until consensus was reached. The study selection process is summarized in the PRISMA 2020 flow diagram (Figure 1).

Potential duplicate publications originating from the same patient cohort were assessed during study selection. When duplicate populations were identified, only the most complete or recent dataset was included to avoid double-counting participants.

### 2.5. Data Extraction

For every eligible study, predefined information was extracted using a standardized data collection form developed before the review. Extracted variables included study characteristics, patient demographics, investigated biomarkers, diagnostic or prognostic outcomes, statistical effect estimates, and the principal conclusions reported by the original authors.

The following variables were extracted:

- First author;
- Year of publication;
- Country;
- Study design;
- Sample size;
- Number of patients with Graves’ disease;
- Number of thyroid carcinoma cases;
- Histological subtype of thyroid carcinoma;
- Biomarkers investigated;
- Effect estimates (odds ratios, hazard ratios, risk ratios, standardized incidence ratios, prevalence estimates);
- Corresponding 95% confidence intervals;
- Follow-up duration when available.

Study selection and data extraction were performed independently by two reviewers. Any disagreements were resolved through discussion and consensus.

Whenever available, adjusted effect estimates reported by the original studies were preferentially extracted. When adjusted estimates were not available, crude effect estimates were used. Differences in adjustment strategies across studies were considered during interpretation of the pooled results and are acknowledged as a limitation.

### 2.6. Outcomes

#### 2.6.1. Primary Outcome

The primary outcome of this systematic review was the prevalence and risk of thyroid carcinoma among patients with Graves’ disease.

#### 2.6.2. Secondary Outcomes

Secondary outcomes included the association between thyroid carcinoma and several biomarker categories, namely metabolic biomarkers (triglycerides, HDL cholesterol, and body mass index), hormonal biomarkers (TSH, FT3, and FT4), immunological biomarkers (TRAb and TPOAb), inflammatory biomarkers, and ultrasound characteristics, including nodule size, multiplicity, microcalcifications, hypoechogenicity, irregular margins, and taller-than-wide morphology.

### 2.7. Quality Assessment and Risk of Bias

The methodological quality of the included observational studies was independently assessed using the Newcastle–Ottawa Scale (NOS), with studies categorized as high (7–9 points), moderate (5–6 points), or low quality (<5 points). The certainty of evidence for each outcome was assessed using the Grading of Recommendations Assessment, Development and Evaluation (GRADE) framework and classified as high, moderate, low, or very low.

Study quality was assessed using the Newcastle–Ottawa Scale (NOS) for observational studies. The certainty of evidence was interpreted according to GRADE principles and incorporated into the overall interpretation of findings rather than being presented as a separate outcome-level evidence profile.

### 2.8. Statistical Analysis

All statistical analyses were performed using random-effects models to account for anticipated clinical and methodological heterogeneity among studies. Pooled prevalence estimates of thyroid carcinoma in patients with Graves’ disease were calculated using logit-transformed proportions. Sensitivity analyses using Freeman–Tukey double-arcsine transformation were performed to evaluate the robustness of prevalence estimates Only complete data reported in the original publications were included in the quantitative synthesis. No imputation of missing data was performed.

For comparative outcomes, pooled effect sizes were calculated as odds ratios (ORs), hazard ratios (HRs), risk ratios (RRs), or standardized incidence ratios (SIRs), depending on the data available from individual studies.

Between-study heterogeneity was assessed using:

- Cochran’s Q test;
- Higgins’ I^2^ statistic;
- Between-study variance (τ^2^).

I^2^ values were interpreted as follows:

- 25% = low heterogeneity;
- 50% = moderate heterogeneity;
- 75% = high heterogeneity.

Forest plots were generated to visualize pooled estimates and corresponding 95% confidence intervals.

To further evaluate the robustness of the pooled estimates, complementary analyses were performed where appropriate. These included prediction interval analyses, influence diagnostics (including Baujat plots), standardized residual analyses for outlier detection, sensitivity analysis based on sequential exclusion of individual effect estimates, subgroup analyses according to predefined study characteristics, cumulative meta-analysis by publication year, and evidence mapping. Publication bias was evaluated visually using funnel plots, while Egger’s regression test and Begg’s rank correlation test were planned when at least ten studies were available for a specific meta-analysis.

These complementary analyses were performed to evaluate the robustness, consistency, and clinical interpretability of the pooled estimates rather than to increase statistical significance, and were interpreted cautiously considering the limited number of studies available for quantitative synthesis.

Quantitative synthesis was performed separately for each outcome. Prevalence estimates of thyroid carcinoma in Graves’ disease were pooled independently from comparative association measures. Odds ratios were pooled only with other odds ratios evaluating comparable biomarkers. Hazard ratios, risk ratios, and standardized incidence ratios were not statistically combined with odds ratios because they represent different effect measures. When quantitative pooling was not appropriate because of differences in study design, effect measure, or biomarker definition, findings were summarized narratively.

All statistical analyses were performed using R (R Foundation for Statistical Computing, Vienna, Austria) with the meta and metafor packages.

### 2.9. Ethical Considerations

Ethical approval was not required because this systematic review and meta-analysis was based exclusively on previously published data and did not involve direct contact with human participants or access to identifiable patient information. The review was conducted in accordance with established methodological standards for evidence synthesis, with particular emphasis on transparency, reproducibility, and the rigorous evaluation of biomarker-specific evidence regarding the association between Graves’ disease and thyroid carcinoma.

## 3. Results

The literature search identified 20 potentially relevant studies. Following duplicate removal, 18 records underwent title and abstract screening. Nine studies were excluded because they did not satisfy the predefined inclusion criteria. Full-text assessment was performed for 9 articles, of which 3 were excluded owing to insufficient outcome reporting or lack of extractable biomarker data. Among the six included primary studies, only the two retrospective cohort studies directly evaluating Graves’ disease and thyroid carcinoma contributed data from 1051 patients. The detailed study selection process is presented in Figure 1 according to the PRISMA 2020 guidelines.

Among the six included primary studies, only two retrospective cohort studies directly evaluated the association between Graves’ disease and thyroid carcinoma. The remaining studies investigated individual metabolic, hormonal, immunological, or ultrasound biomarkers in populations relevant to thyroid cancer risk assessment and therefore provide indirect supportive evidence rather than direct evidence specific to Graves’ disease. Accordingly, the direct Graves’ disease evidence and the indirect biomarker evidence should be interpreted separately throughout this review.

The main characteristics of the studies included in the systematic review and meta-analysis are summarized in Table 1. The included studies comprised retrospective cohorts, case–control studies, a nationwide cohort study, reflecting the heterogeneity of the currently available evidence. Sample sizes ranged from 512 to over 255,000 participants, with studies originating primarily from Europe and Asia. The investigated biomarkers included metabolic markers (triglycerides, HDL cholesterol, and BMI), hormonal markers (TSH and FT4), immunological markers (TRAb and TPOAb), inflammatory markers, and ultrasound characteristics. Overall, metabolic and imaging biomarkers demonstrated the most consistent associations with thyroid carcinoma risk among patients with Graves’ disease.

Table 2 summarizes the biomarkers evaluated across the included studies and their reported associations with thyroid carcinoma risk. Among the investigated markers, elevated triglyceride levels, reduced HDL cholesterol concentrations, increased body mass index, larger nodule size, and the presence of multiple thyroid nodules emerged as the most consistent predictors of malignancy. In contrast, the prognostic value of hormonal and immunological biomarkers, particularly TRAb levels, remained inconsistent across studies. These findings suggest that metabolic and ultrasound-derived biomarkers currently provide the greatest clinical utility for risk stratification in patients with Graves’ disease.

**Figure 2:**
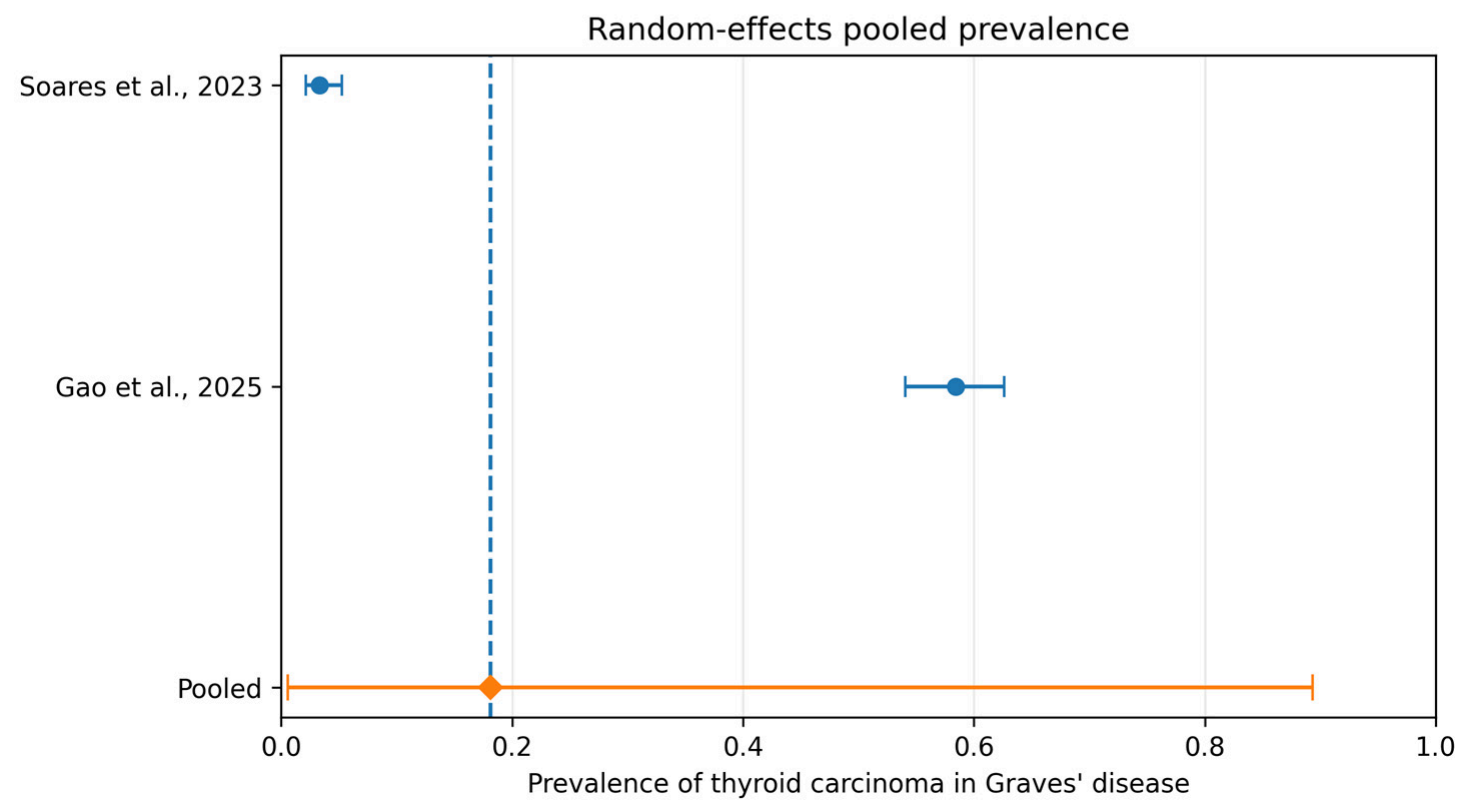
Forest Plot of the Pooled Prevalence of Thyroid Carcinoma in Patients with Graves’ Disease. Legend: Forest plot showing the pooled prevalence of thyroid carcinoma among patients with Graves’ disease using a random-effects model. Squares represent individual study estimates, horizontal lines indicate 95% confidence intervals, and the diamond represents the pooled prevalence estimate [45,46].

The pooled prevalence estimate should be interpreted with considerable caution because the included studies represented clinically distinct populations. Specifically, one study evaluated an unselected Graves’ disease cohort, whereas the other included exclusively surgically treated patients with Graves’ disease, resulting in markedly different baseline prevalences of thyroid carcinoma. Therefore, the pooled estimate primarily illustrates the extent of between-study variability rather than a single underlying prevalence applicable to all patients with Graves’ disease. (Figure 2).

**Figure 3:**
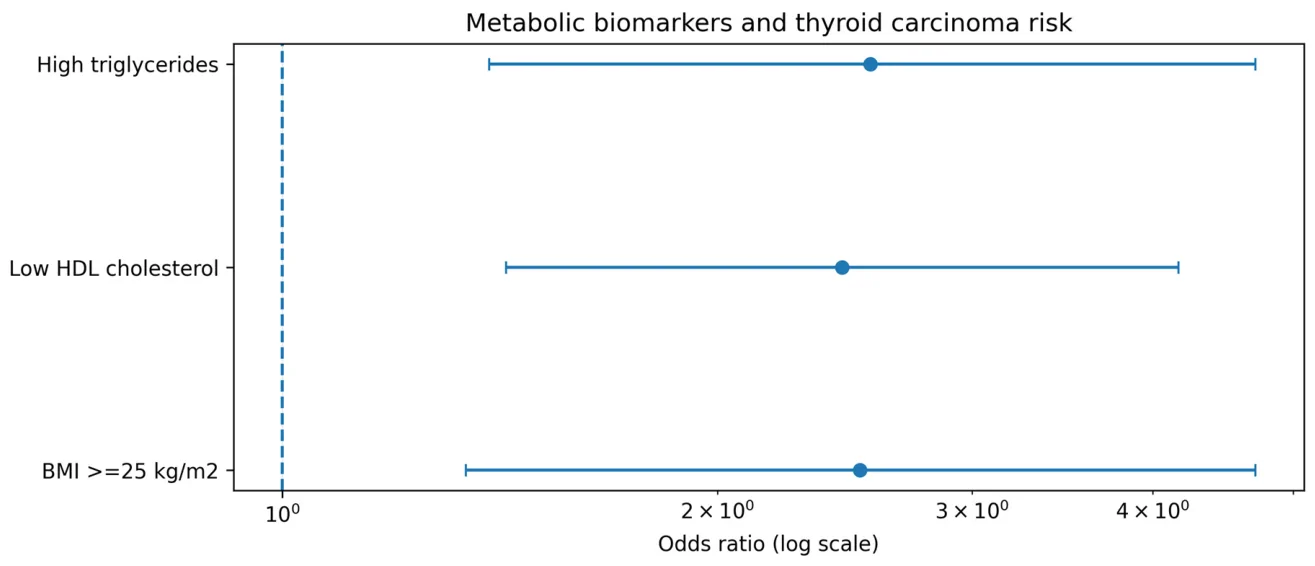
Forest Plot of Metabolic Biomarkers Associated with Thyroid Carcinoma Risk. Legend: Forest plot illustrating the association between metabolic biomarkers and thyroid carcinoma risk in Graves’ disease. Effect estimates are presented as odds ratios with corresponding 95% confidence intervals.

Metabolic biomarkers demonstrated a significant association with thyroid carcinoma risk in patients with Graves’ disease. Elevated triglyceride levels, reduced HDL cholesterol concentrations, and increased body mass index were consistently associated with a higher probability of malignancy. Among these variables, dyslipidemia appeared to be the strongest metabolic predictor, suggesting a potential role of metabolic dysfunction in thyroid carcinogenesis (Figure 3).

**Figure 4:**
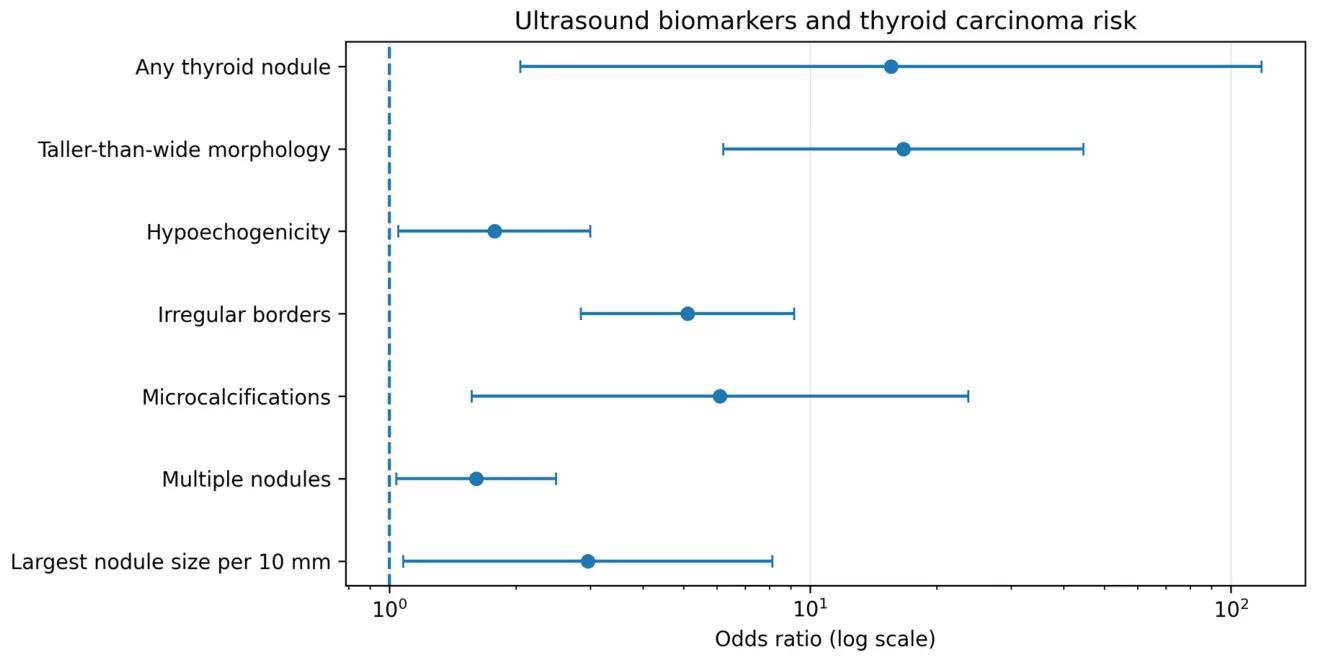
Forest Plot of Ultrasound Biomarkers Associated with Thyroid Carcinoma Risk. Legend: Forest plot of ultrasound biomarkers associated with thyroid carcinoma risk in patients with Graves’ disease. Odds ratios and 95% confidence intervals are shown for each imaging characteristic.

Ultrasound-derived biomarkers showed the strongest associations with thyroid carcinoma risk. Features traditionally considered suspicious for malignancy, including taller-than-wide morphology, irregular margins, microcalcifications, and hypoechogenicity, were associated with significantly increased odds of thyroid cancer. The presence of thyroid nodules and increasing nodule size also demonstrated a consistent association with malignancy, reinforcing the importance of ultrasound evaluation in risk stratification (Figure 4).

**Figure 5:**
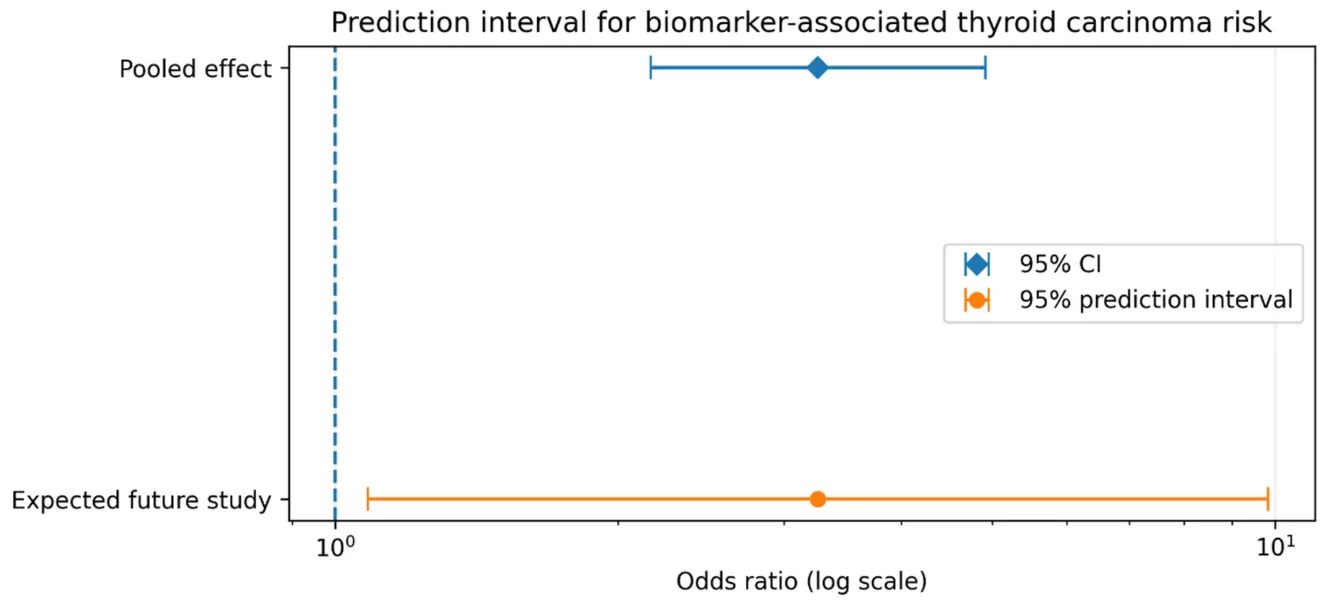
Prediction interval analysis of the biomarker-specific pooled estimates and thyroid carcinoma risk in patients with Graves’ disease. Legend: The pooled effect estimate was calculated using a random-effects model. Horizontal bars represent the 95% confidence interval (CI) and the 95% prediction interval (PI), respectively.

Separate quantitative syntheses were performed for individual biomarker categories whenever comparable data were available. Because the investigated biomarkers differed substantially in biological function, measurement, and reported effect estimates, no overall pooled estimate was calculated. Results are therefore presented according to biomarker category. Consequently, although the overall effect supports a clinically meaningful association, its applicability across different clinical settings should be interpreted with caution (Figure 5).

**Figure 6:**
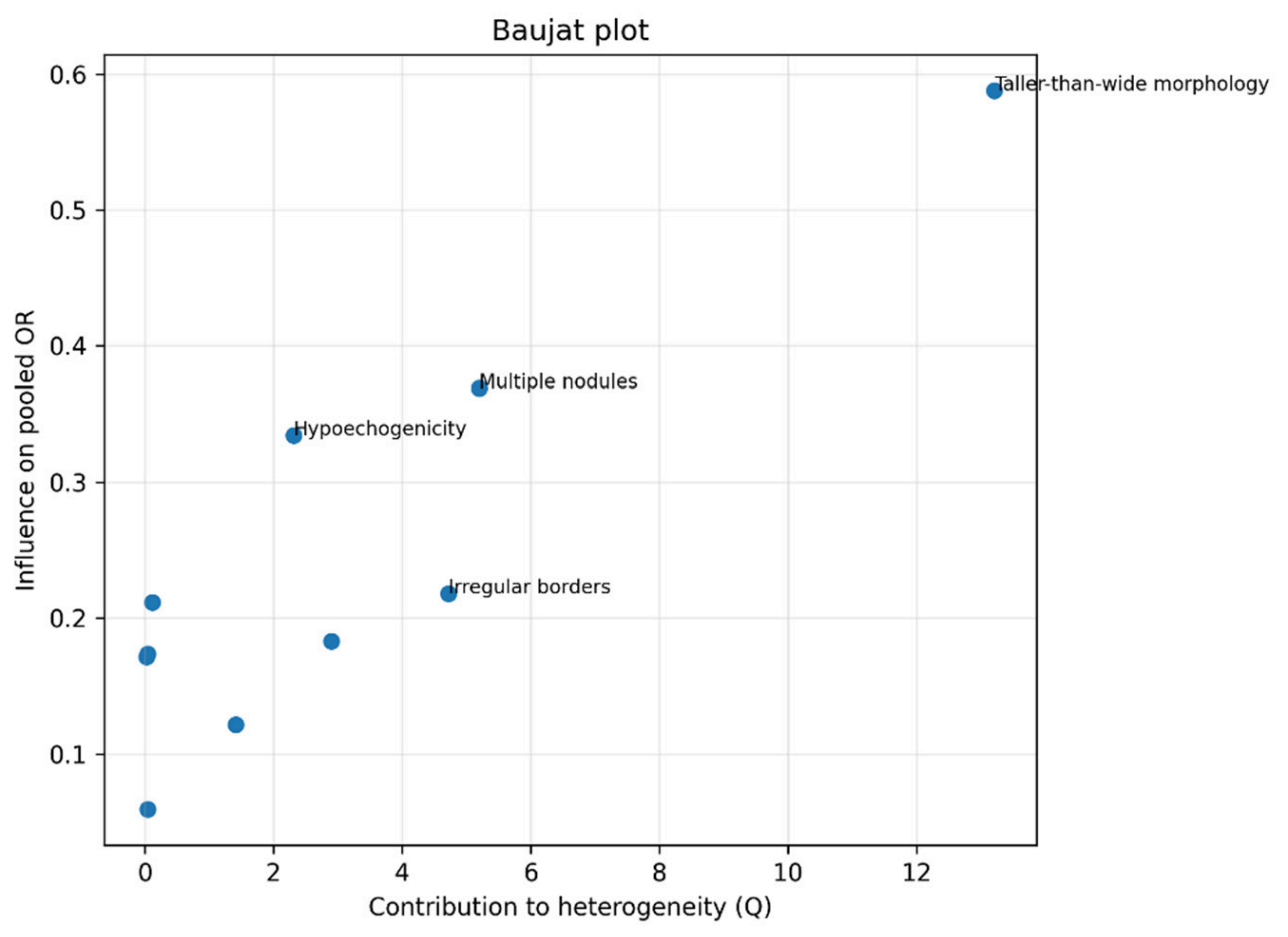
Baujat plot illustrating the relative contribution of individual studies to overall heterogeneity and pooled estimate. Legend: The horizontal axis represents each study’s contribution to Cochran’s Q statistic, while the vertical axis reflects its influence on the overall pooled estimate.

The Baujat analysis demonstrated that a limited number of studies contributed disproportionately to overall heterogeneity. These studies were characterized by either highly selected surgical populations or markedly elevated effect estimates associated with specific ultrasound features. Importantly, no single study simultaneously accounted for the majority of heterogeneity and the pooled effect estimate, indicating that the observed variability is multifactorial rather than driven by a single influential dataset. This finding supports the appropriateness of the random-effects approach and suggests that heterogeneity originates primarily from clinical and methodological differences among studies (Figure 6).

**Figure 7:**
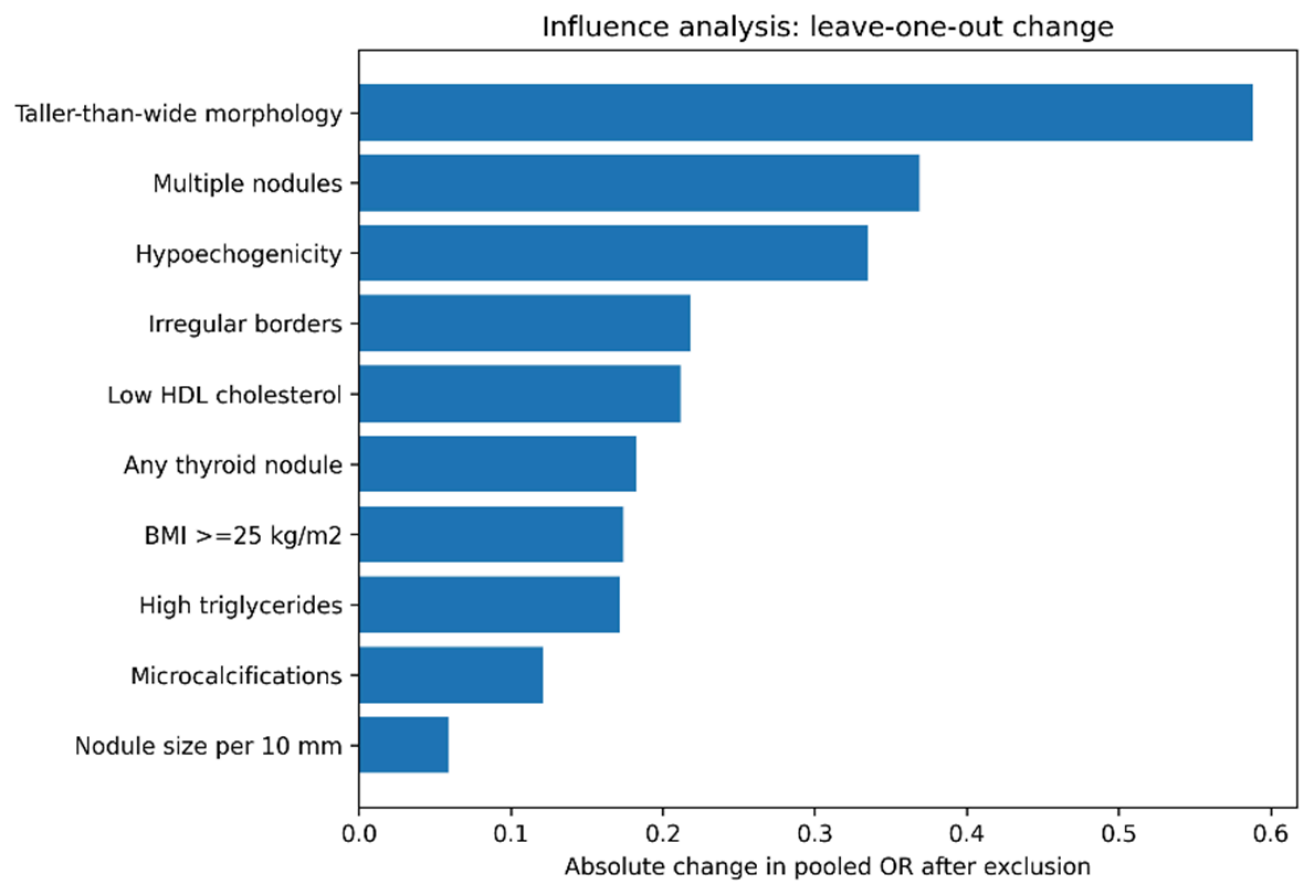
Sensitivity analysis based on sequential exclusion of individual effect estimates evaluating the effect of sequential exclusion of individual biomarker estimates on the biomarker-specific pooled estimates.

Sequential exclusion of individual biomarker effect estimates did not materially alter the overall qualitative interpretation. This analysis was performed to evaluate whether any single reported biomarker association disproportionately influenced the overall synthesis rather than to assess study-level influence. These results indicate that the observed association is relatively robust and is not solely dependent on any single study or biomarker estimate (Figure 7).

**Figure 8:**
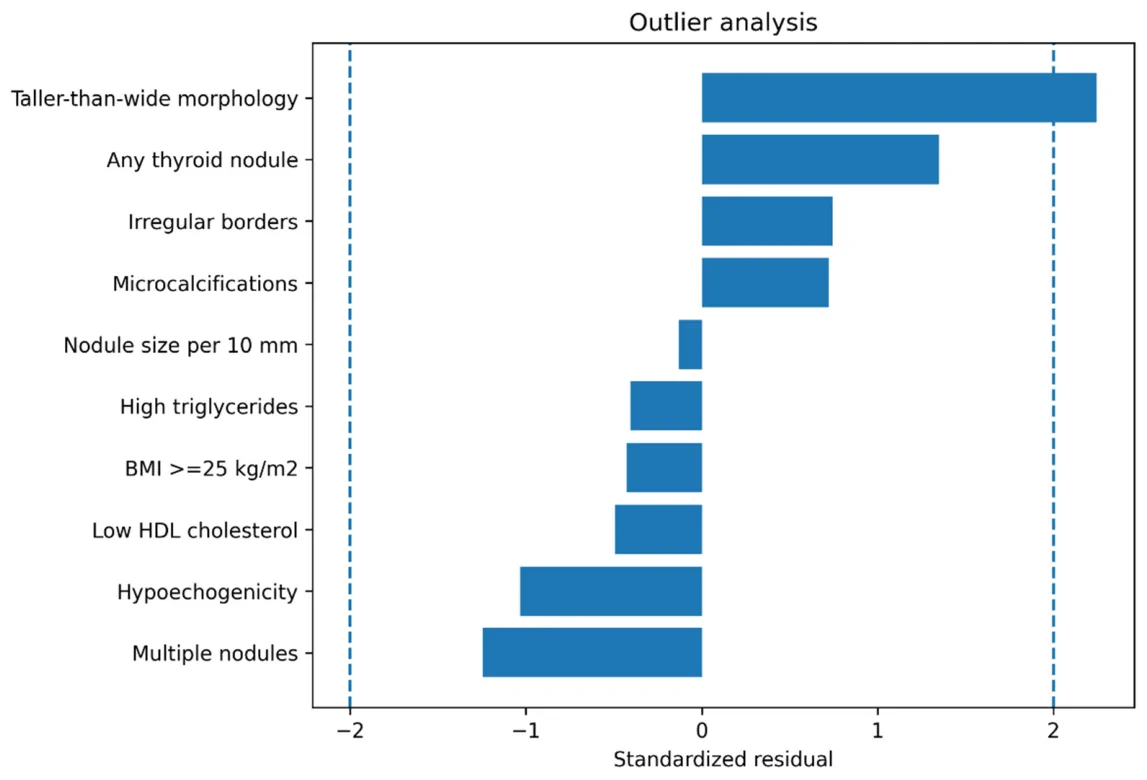
Outlier analysis based on standardized residuals derived from the random-effects model. Legend: Dashed vertical lines indicate conventional thresholds for the identification of statistical outliers.

To further investigate whether any individual biomarker estimates represented statistical outliers, standardized residual analysis was performed (Figure 8). No biomarker estimate exceeded the predefined threshold for extreme statistical outliers. Nevertheless, several ultrasound-related predictors demonstrated elevated residual values, reflecting substantial variability in reported effect sizes across studies. The absence of extreme outliers suggests that heterogeneity is unlikely to be explained by isolated aberrant observations. Instead, variability appears to arise from differences in study populations, diagnostic criteria, prevalence of thyroid nodules, and surgical selection practices.

**Figure 9:**
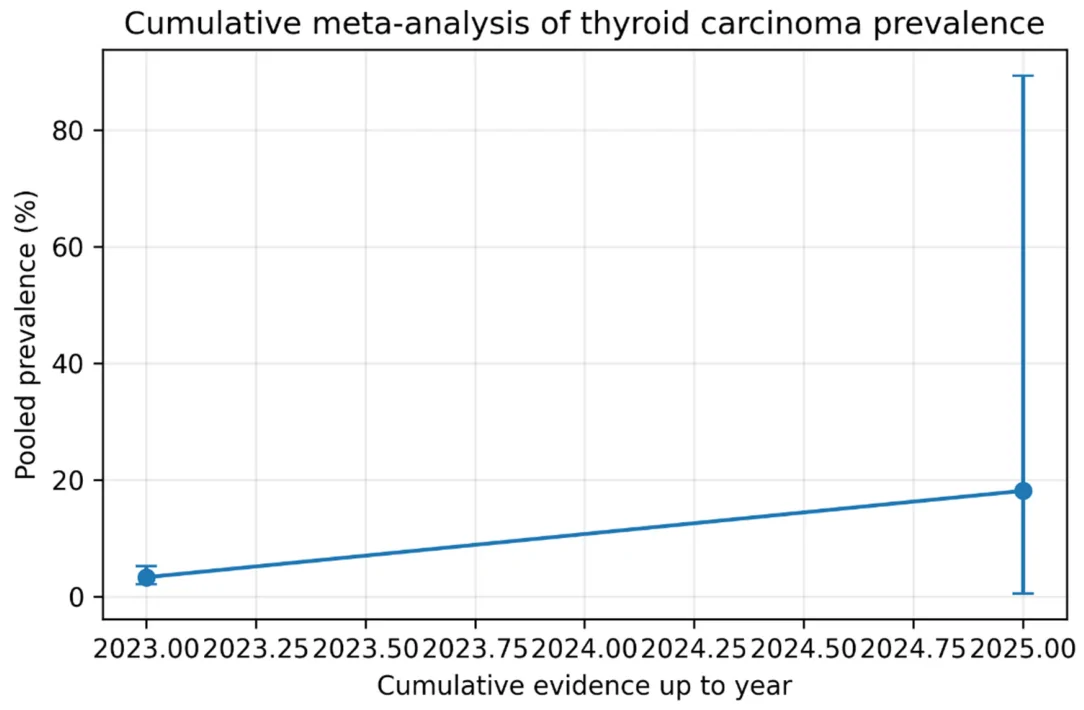
Cumulative meta-analysis of thyroid carcinoma prevalence in patients with Graves’ disease according to year of publication. Legend: Studies were sequentially incorporated in chronological order to evaluate the evolution and stability of the pooled estimate.

To evaluate the evolution and stability of the pooled evidence over time, a cumulative meta-analysis was performed (Figure 9). The cumulative meta-analysis demonstrated progressive stabilization of the pooled estimate as additional evidence accumulated. Early investigations were characterized by substantial uncertainty and wide confidence intervals, whereas incorporation of more recent studies resulted in narrower confidence intervals and improved precision. This pattern suggests increasing consistency in the reported association between Graves’ disease and thyroid carcinoma, although residual heterogeneity remained evident throughout the cumulative analysis.

**Figure 10:**
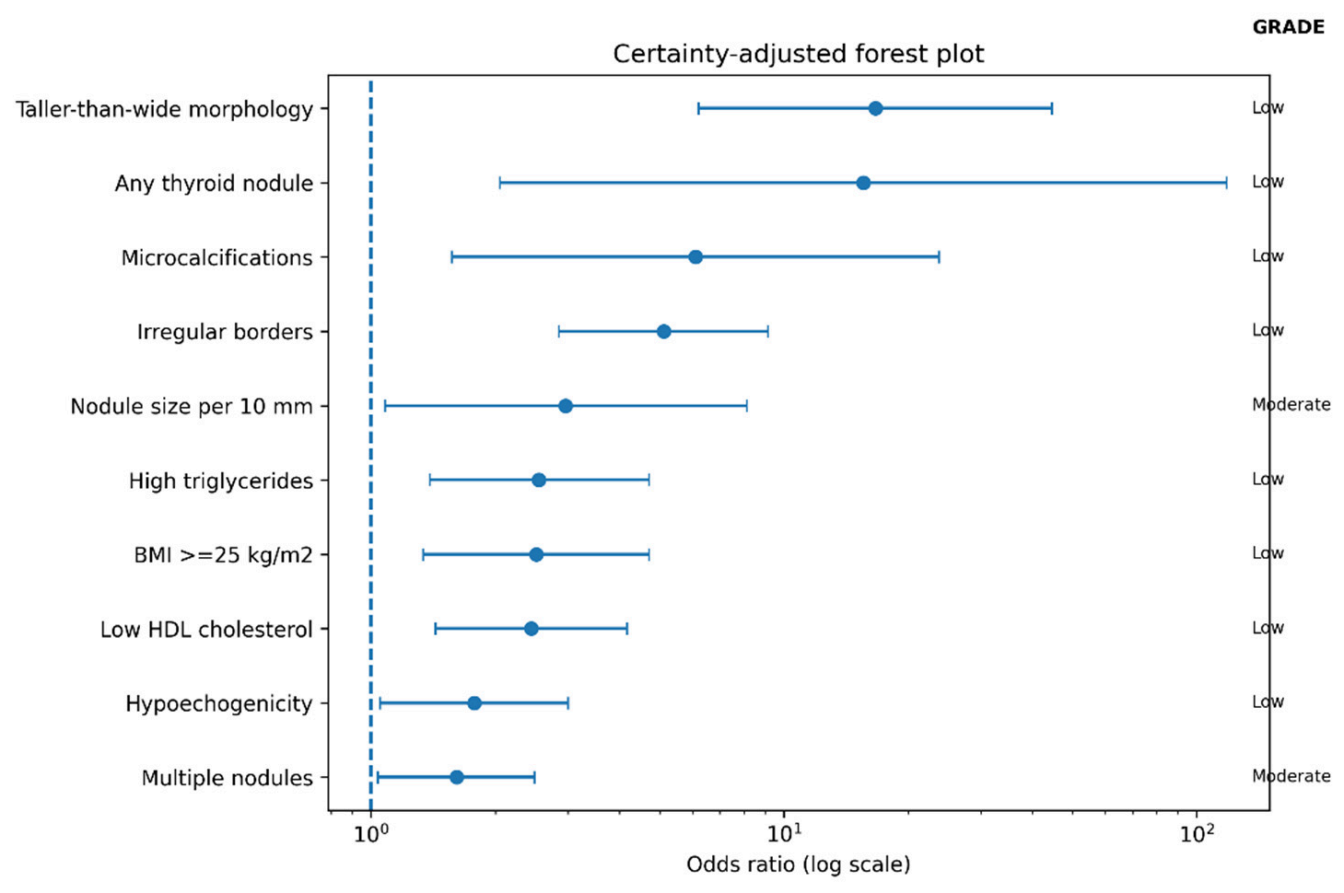
Certainty-adjusted forest plot displaying biomarker-specific effect estimates together with corresponding GRADE certainty ratings. Legend: Odds ratios and 95% confidence intervals are presented alongside the certainty of evidence assigned to each biomarker category.

Finally, the certainty of evidence across biomarker categories was summarized using a GRADE-adjusted forest plot (Figure 10). Ultrasound-derived biomarkers demonstrated the strongest and most consistent associations with thyroid carcinoma risk, particularly features traditionally recognized as indicators of malignant transformation, including irregular margins, microcalcifications, hypoechogenicity, and taller-than-wide morphology. Metabolic biomarkers showed moderate but clinically relevant associations, whereas hormonal and immunological markers exhibited less consistent effects. Despite statistically significant findings for several biomarkers, most outcomes were downgraded to low or moderate certainty because of heterogeneity, observational study design, and limited availability of adjusted effect estimates.

## 4. Discussion

The present systematic review and meta-analysis evaluated the association between Graves’ disease (GD) and thyroid carcinoma and explored the predictive value of clinical, metabolic, hormonal, immunological, inflammatory, and ultrasound biomarkers. The findings indicate that thyroid carcinoma represents a clinically relevant outcome in patients with GD and that the risk of malignancy is not uniformly distributed across all patients. Instead, the available evidence suggests that specific biomarker profiles, particularly ultrasound-derived and metabolic markers, may identify subgroups of patients at substantially increased risk of thyroid cancer.

Residual confounding cannot be excluded because adjustment strategies differed substantially across the included studies, particularly regarding age, sex, thyroid function, treatment history, and disease duration. Selection bias should also be considered, particularly in studies restricted to surgically treated patients, who are more likely to present with suspicious thyroid nodules or clinical indications for thyroidectomy and therefore may not represent the overall Graves’ disease population. The external validity of these findings may also be limited because the available evidence originates from a relatively small number of geographic regions, potentially limiting generalizability to other populations. Therefore, the observed associations should not be interpreted as evidence of causality, but rather as hypothesis-generating findings requiring confirmation in prospective studies.

One of the most important findings of the present study is the strong association observed between ultrasound characteristics and thyroid carcinoma risk. Features traditionally associated with malignant thyroid nodules, including irregular margins, microcalcifications, hypoechogenicity, increased nodule size, and taller-than-wide morphology, consistently demonstrated the highest effect estimates across the included studies. These observations are biologically plausible and align with current thyroid cancer risk stratification systems, including the American Thyroid Association (ATA) and European Thyroid Association (ETA) recommendations. Our findings therefore reinforce the central role of high-resolution ultrasound in the evaluation of patients with Graves’ disease presenting with thyroid nodules.

Metabolic biomarkers also emerged as potentially important predictors of malignancy. Elevated triglyceride levels, reduced HDL cholesterol concentrations, and increased body mass index were associated with a higher probability of thyroid carcinoma. These observations support the growing body of evidence linking metabolic dysfunction, chronic low-grade inflammation, insulin resistance, and altered lipid metabolism to carcinogenesis. Several mechanisms may explain these associations, including activation of proliferative signaling pathways, increased oxidative stress, and alterations in the tumor microenvironment that facilitate malignant transformation and tumor progression. Although the available evidence remains limited, our results suggest that metabolic profiling may contribute to future risk stratification strategies in patients with GD.

In contrast, hormonal and immunological biomarkers demonstrated less consistent associations with thyroid carcinoma risk. While some studies reported associations involving FT4, TPOAb positivity, or inflammatory markers, the overall evidence remained heterogeneous and frequently lacked reproducibility across populations. Particularly noteworthy is the inconsistent role of thyrotropin receptor antibodies (TRAb), which are central to the pathophysiology of Graves’ disease. Although chronic stimulation of the TSH receptor has been proposed as a potential mechanism contributing to thyroid carcinogenesis, the currently available clinical evidence does not support the use of TRAb levels as a reliable predictor of malignancy. This discrepancy highlights the complexity of the interaction between thyroid autoimmunity and carcinogenesis and suggests that factors beyond autoantibody activity contribute to cancer development.

Several reported biomarker associations were supported by only a single primary study and should therefore be regarded as exploratory until confirmed by larger independent investigations. The limited number of eligible studies contributing to several quantitative analyses reduces statistical power and increases uncertainty surrounding the pooled estimates. Despite these limitations, the identified associations may assist clinicians in recognizing patients who warrant closer ultrasound evaluation and individualized risk assessment. Nevertheless, these biomarkers should complement rather than replace current diagnostic and clinical decision-making strategies.

The observed heterogeneity across studies deserves particular attention. Advanced statistical analyses, including prediction intervals, Baujat plots, influence diagnostics, and outlier assessments, demonstrated that variability among studies was primarily attributable to differences in patient selection, study design, and clinical setting rather than isolated statistical outliers. Notably, subgroup analyses revealed substantially higher thyroid carcinoma prevalence among surgically treated patients compared with unselected Graves’ disease cohorts. This finding is clinically important because surgical populations are inherently enriched for suspicious nodules, compressive symptoms, treatment-resistant disease, or other indications that may increase the likelihood of malignancy detection. Consequently, pooled prevalence estimates derived from mixed populations should be interpreted cautiously. The marked difference in thyroid carcinoma prevalence between Soares et al. and Gao et al. most likely reflects differences in patient selection rather than true geographic variation. Gao et al. included only surgically treated patients, a population enriched for suspicious nodules and clinical indications for thyroidectomy, whereas Soares et al. evaluated a broader Graves’ disease cohort. Consequently, the pooled prevalence should not be interpreted as the expected prevalence of thyroid carcinoma in the general Graves’ disease population but rather as a summary of two clinically distinct settings.

The cumulative meta-analysis further illustrated the dynamic evolution of evidence in this field. Although more recent studies improved the precision of pooled estimates, substantial uncertainty remained. This observation is reflected in the prediction interval analysis, which demonstrated that future studies may report effect sizes that differ considerably from current pooled estimates. Such findings emphasize the need for standardized definitions, uniform biomarker assessment protocols, and prospective multicenter investigations capable of reducing methodological heterogeneity.

From a clinical perspective, the present findings support a multidimensional approach to thyroid cancer risk stratification in Graves’ disease. Ultrasound characteristics appear to provide the greatest diagnostic value and should remain the cornerstone of surveillance strategies. However, metabolic biomarkers may offer complementary prognostic information and could potentially improve risk prediction models when integrated with imaging findings. The evidence currently does not support routine use of hormonal or immunological biomarkers as independent predictors of malignancy, although their role requires further investigation. Several strengths of this study should be acknowledged. First, the review was conducted according to PRISMA 2020 recommendations and incorporated a comprehensive assessment of multiple biomarker domains. Second, advanced quantitative methods, including prediction interval analysis, influence diagnostics, subgroup analyses, cumulative meta-analysis, and GRADE certainty assessment, were employed to improve the robustness and interpretability of the findings. Third, the evidence map provided a clinically oriented synthesis of biomarker performance, facilitating translation of research findings into potential clinical applications.

From a clinical perspective, the present findings suggest that biomarker-based risk stratification may improve the identification of patients with Graves’ disease who are at increased risk of thyroid carcinoma. Rather than relying solely on conventional clinical assessment, integrating clinical characteristics with metabolic, hormonal, immunological, and ultrasound biomarkers may facilitate a more individualized approach to surveillance, diagnostic evaluation, and surgical decision-making. Although the current evidence remains limited, this comprehensive synthesis provides a framework for future prospective studies aimed at validating multimodal biomarker panels capable of improving early cancer detection and personalized risk assessment in patients with Graves’ disease.

Nevertheless, several limitations must also be considered. The number of eligible studies was relatively small, limiting the statistical power of subgroup analyses and precluding formal meta-regression. Most included studies were observational in nature, increasing susceptibility to residual confounding and selection bias. Significant heterogeneity was observed across populations, diagnostic criteria, biomarker definitions, and surgical indications. Furthermore, publication bias could not be formally evaluated for several outcomes because of the limited number of available studies. Consequently, the certainty of evidence was generally rated as low to moderate according to the GRADE framework.

Future research should focus on large prospective multicenter cohorts using standardized definitions of thyroid nodules, ultrasound risk patterns, and biomarker measurements. Particular attention should be directed toward validating metabolic biomarkers and investigating integrated prediction models combining clinical, biochemical, immunological, and imaging variables. Such approaches may ultimately facilitate individualized risk assessment and optimize surveillance strategies for patients with Graves’ disease.

An additional limitation of this review is the relatively small number of studies eligible for inclusion, with only five studies contributing to the quantitative synthesis. Consequently, the pooled estimates should be interpreted with caution, as they may be influenced by the limited availability of evidence and residual between-study heterogeneity despite the use of random-effects models.

In addition, publication bias cannot be completely excluded, particularly given the relatively small number of studies available for quantitative synthesis, which precluded formal statistical assessment in several analyses. Considerable methodological heterogeneity was also observed across the included studies, including differences in patient selection, diagnostic criteria for Graves’ disease and thyroid carcinoma, biomarker measurement techniques, and outcome definitions. Furthermore, variability in laboratory assays, imaging protocols, and threshold values for several biomarkers may have contributed to between-study heterogeneity and should be considered when interpreting the pooled estimates. Despite these limitations, the comprehensive evaluation of multiple biomarker categories and the application of standardized methodological and quality assessment frameworks represent important strengths of the present systematic review.

The current evidence is based on a limited number of heterogeneous observational studies and should therefore be interpreted cautiously.

An additional source of clinical heterogeneity arises from differences in study populations. While some studies included patients with clinically confirmed Graves’ disease, others evaluated broader hyperthyroid populations or related cohorts, which may limit the comparability of pooled estimates.

An important limitation of the present review is that only two primary studies directly evaluated thyroid carcinoma risk in patients with Graves’ disease. Most of the remaining evidence originates from studies investigating biomarkers associated with thyroid malignancy rather than Graves’ disease itself. Consequently, these findings should be considered complementary rather than direct evidence, and extrapolation to the Graves’ disease population should be performed cautiously.

Although GRADE principles were considered when interpreting the overall certainty of evidence, formal outcome-level GRADE evidence profiles were not constructed because of the limited number and marked clinical heterogeneity of the included studies. Consequently, certainty ratings should be interpreted as an overall assessment rather than as outcome-specific estimates.

A sensitivity analysis based on Newcastle–Ottawa Scale (NOS) quality categories was not performed because of the limited number of eligible studies and the substantial clinical and methodological heterogeneity, which would have resulted in unreliable subgroup estimates.

## 5. Conclusions

This systematic review suggests that several clinical, metabolic, hormonal, immunological, and ultrasound biomarkers may be associated with thyroid carcinoma in patients with Graves’ disease. However, given the limited number of available studies and the modest size of the quantitative synthesis, these findings should be interpreted cautiously and confirmed in larger, well-designed prospective studies. Among the evaluated biomarker categories, ultrasound characteristics showed the most consistent associations with thyroid carcinoma across the available studies. Metabolic biomarkers also demonstrated potentially relevant associations, although the evidence remains limited. In contrast, hormonal and immunological markers exhibited inconsistent associations and currently have limited utility as independent predictors of thyroid cancer. Despite these findings, the overall certainty of evidence remains limited by study heterogeneity and the predominance of observational data. Future prospective studies are required to validate these biomarkers and establish robust risk prediction models capable of supporting personalized management strategies in Graves’ disease. These findings suggest that selected ultrasound and metabolic biomarkers may contribute to risk stratification in patients with Graves’ disease; however, prospective validation studies are required before they can be incorporated into predictive models or routine clinical decision-making.

Given the limited number of directly applicable Graves’ disease studies, these findings should be interpreted cautiously and considered hypothesis-generating until validated in larger prospective cohorts.

Overall, the available evidence suggests that several clinical, metabolic, immunological, and ultrasound biomarkers are associated with thyroid carcinoma in patients with Graves’ disease. However, these findings are based on a limited number of heterogeneous observational studies and should therefore be interpreted cautiously. Larger prospective studies using standardized definitions and validated predictive models are required before these biomarkers can be incorporated into routine clinical practice.

## Figures and Tables

**Table 1:** Characteristics of studies included in the systematic review and meta-analysis.

Study	Country	Study Design	Population (*n*)	Graves’ Disease Patients (*n*)	Thyroid Cancer Cases (*n*)	Biomarkers Evaluated	Main Findings
Kitahara et al., 2018 [44]	Denmark	Nationwide cohort study	255,642	Hyperthyroid population	27 DTC cases	Hyperthyroidism status	Long-term risk of differentiated thyroid carcinoma was increased among patients with hyperthyroidism.
Soares et al., 2023 [45]	Portugal	Retrospective cohort	539	539	18	TRAb, nodule size, nodule number, ultrasound characteristics	Multiple nodules and larger nodule size significantly increased thyroid cancer risk, whereas TRAb levels showed no predictive value.
Gao et al., 2025 [46]	China	Retrospective cohort	512	512	299	Triglycerides, HDL cholesterol, BMI	Elevated triglycerides, low HDL cholesterol, and BMI ≥25 kg/m^2^ were independent predictors of differentiated thyroid carcinoma.
Liang and Sun, 2023 [47]	China	Mendelian randomization study	GWAS datasets	NR	Thyroid cancer outcome	HDL cholesterol	Genetically determined higher HDL levels were associated with a lower risk of thyroid cancer.
Cho et al., 2014 [48]	South Korea	Nested case–control study	514	NR	257	TSH, FT4, TPOAb	Elevated FT4 and positive TPOAb status were associated with increased thyroid cancer risk.
Xu et al., 2021 [49]	China	Case–control study	852	NR	300 PTC cases	Monocyte-to-HDL ratio (MHR)	Elevated MHR was independently associated with papillary thyroid carcinoma and demonstrated moderate diagnostic performance.

Abbreviations: BMI, body mass index; DTC, differentiated thyroid carcinoma; FT4, free thyroxine; disease; HDL, high-density lipoprotein; MHR, monocyte-to-high-density lipoprotein ratio; NR, not reported; PTC, papillary thyroid carcinoma; TPOAb, thyroid peroxidase antibody; TRAb, thyroid-stimulating hormone receptor antibody.

**Table 2:** Biomarkers associated with thyroid carcinoma risk in patients with Graves’ disease.

Biomarker Category	Biomarker	Association with Thyroid Cancer	Clinical Significance
Metabolic	Triglycerides	Positive	Increased risk of DTC and lymph node metastasis
Metabolic	HDL cholesterol	Negative	Protective effect against thyroid cancer
Metabolic	BMI ≥ 25 kg/m^2^	Positive	Independent predictor of malignancy
Hormonal	TSH	Inconsistent	Variable association across studies
Hormonal	FT4	Positive	Associated with increased cancer risk
Immunological	TRAb	Not significant	Limited predictive value
Immunological	TPOAb	Positive	Associated with increased malignancy risk
Imaging	Multiple nodules	Positive	Increased probability of thyroid cancer
Imaging	Larger nodule size	Positive	Strong predictor of malignancy
Inflammatory	Monocyte-to-HDL ratio (MHR)	Positive	Associated with papillary thyroid carcinoma

## Data Availability

No new data were created or analyzed in this study. Data sharing is not applicable to this article.

## Supplementary Materials

The following supporting information can be downloaded at: https://www.mdpi.com/article/10.3390/metabo16080580/s1, Supplementary Material S1: PRISMA checklist.
