# Supplementary material for: Association Between Graves’ Disease and the Risk of Thyroid Carcinoma: The Role of Clinical, Metabolic, Hormonal, Immunological, and Ultrasound Biomarkers
Source: Metabolites. 2026 Aug 17;16(8):580. doi: 10.3390/metabo16080580 (PMC13515884; doi:10.3390/metabo16080580)
Supplement: Supplementary file 1 [file metabolites-16-00580-s001.zip › metabolites-4433774-Supplementary Material S1.pdf]

Section	Item	PRISMA 2020 Checklist Item	Page(s) in manuscript
**TITLE**	1	Identify the report as a systematic review and meta-analysis	**1**
**ABSTRACT**	2	Structured abstract	**Not included** *(should be added before submission)*
**INTRODUCTION**	3	Rationale	**1–2**
	4	Objectives	**2**
**METHODS**	5	Eligibility criteria	**3**
	6	Information sources	**2–3**
	7	Search strategy	**2–3** *(full search strategy should be provided as Supplementary Material)*
	8	Selection process	**3**
	9	Data collection process	**3–4**
	10a	Outcomes	**4**
	10b	Other variables	**3–4**
	11	Risk of bias assessment	**4**
	12	Effect measures	**5**
	13a	Selection for synthesis	**5–6**
	13b	Data preparation	**5**
	13c	Methods for tabulation/display	**5–6**
	13d	Statistical synthesis	**5**
	13e	Exploration of heterogeneity	**5–6**
	13f	Sensitivity analyses	**6**
	14	Reporting bias assessment	**6**
	15	Certainty assessment (GRADE)	**4**
**RESULTS**	16a	Search results	**6–7**
	16b	Excluded studies and reasons	**6–7 (Figure 1)**
	17	Characteristics of included studies	**7–9 (Table 1)**
	18	Risk of bias results	**Not reported as a separate table** *(recommended: add NOS table)*
	19	Results of individual studies	**7–10 (Tables 1–2)**
	20a	Summary of studies contributing to syntheses	**6–10**
	20b	Statistical synthesis	**11–17 (Figures 2–10)**
	20c	Heterogeneity investigations	**13–16 (Figures 5–9)**
	20d	Sensitivity analyses	**14–15 (Figures 7–8)**

Section	Item	PRISMA 2020 Checklist Item	Page(s) in manuscript
	21	Reporting bias	**6 (Methods only)** *(Funnel plot not presented)*
	22	Certainty of evidence	**17 (Figure 10)**
**DISCUSSION**	23a	Interpretation	**17–19**
	23b	Limitations of evidence	**19**
	23c	Limitations of review process	**19**
	23d	Implications for practice and research	**18–19**
**OTHER INFORMATION**	24a	Registration	**Not reported**
	24b	Protocol access	**Not reported**
	24c	Protocol amendments	**Not applicable**
	25	Funding	**19**
	26	Competing interests	**20**
	27	Availability of data	**20**

Complete PubMed Search Strategy

(("Graves Disease"[Mesh] OR Graves disease OR hyperthyroidism)

AND

("Thyroid Neoplasms"[Mesh] OR thyroid cancer OR thyroid carcinoma)

AND

(biomarker* OR predictor* OR risk factor* OR ultrasound OR TRAb OR TPOAb OR TSH OR FT3 OR FT4 OR BMI OR HDL OR triglycerides))
